# Optimal vaccine allocation for COVID-19 in the Netherlands: A data-driven prioritization

**DOI:** 10.1371/journal.pcbi.1009697

**Published:** 2021-12-13

**Authors:** Fuminari Miura, Ka Yin Leung, Don Klinkenberg, Kylie E. C. Ainslie, Jacco Wallinga

**Affiliations:** 1 Centre for Infectious Disease Control, National Institute for Public Health and the Environment (RIVM), Bilthoven, the Netherlands; 2 Center for Marine Environmental Studies (CMES), Ehime University, Ehime, Japan; 3 School of Public Health, Imperial College London, London, United Kingdom; 4 MRC Centre for Global Infectious Disease Analysis and Abdul Latif Jameel Institute for Disease and Emergency Analytics, Imperial College London, London, United Kingdom; 5 Department of Biomedical Data Sciences, Leiden University Medical Center (LUMC), Leiden, the Netherlands; Fundação Getúlio Vargas: Fundacao Getulio Vargas, BRAZIL

## Abstract

For the control of COVID-19, vaccination programmes provide a long-term solution. The amount of available vaccines is often limited, and thus it is crucial to determine the allocation strategy. While mathematical modelling approaches have been used to find an optimal distribution of vaccines, there is an excessively large number of possible allocation schemes to be simulated. Here, we propose an algorithm to find a near-optimal allocation scheme given an intervention objective such as minimization of new infections, hospitalizations, or deaths, where multiple vaccines are available. The proposed principle for allocating vaccines is to target subgroups with the largest reduction in the outcome of interest. We use an approximation method to reconstruct the age-specific transmission intensity (the next generation matrix), and express the expected impact of vaccinating each subgroup in terms of the observed incidence of infection and force of infection. The proposed approach is firstly evaluated with a simulated epidemic and then applied to the epidemiological data on COVID-19 in the Netherlands. Our results reveal how the optimal allocation depends on the objective of infection control. In the case of COVID-19, if we wish to minimize deaths, the optimal allocation strategy is not efficient for minimizing other outcomes, such as infections. In simulated epidemics, an allocation strategy optimized for an outcome outperforms other strategies such as the allocation from young to old, from old to young, and at random. Our simulations clarify that the current policy in the Netherlands (i.e., allocation from old to young) was concordant with the allocation scheme that minimizes deaths. The proposed method provides an optimal allocation scheme, given routine surveillance data that reflect ongoing transmissions. This approach to allocation is useful for providing plausible simulation scenarios for complex models, which give a more robust basis to determine intervention strategies.

## Author summary

Vaccination is the key to controlling the ongoing COVID-19 pandemic. In the early stages of an epidemic, there is shortage of vaccines. Here, we propose an algorithm that computes an optimal vaccine distribution among groups for different intervention objective (e.g., minimizing new infections, hospitalizations, or deaths). This method is applicable even when multiple vaccines are available. Our results reveal that an allocation scheme optimized for one specific objective is not necessarily efficient for another, indicating the importance of determining the precise objective in the decision making process at the early phase of distributions. Often, data are scarce in the midst of an epidemics. The proposed method requires routine surveillance data on the number of cases to determine the best possible allocation of vaccines.

## Introduction

SARS-CoV-2 has posed a large threat to public health [1,2]. While non-pharmaceutical interventions (NPIs) reduce transmission [3,4], the societal cost of implementing these measures is enormous [5,6], and the effect is short-lived. Vaccination offers a long-term approach to control COVID-19.

Currently, more than fifteen vaccines have been approved for use, many companies are still conducting clinical trials to develop next generation vaccines [7]. There is a limited amount of vaccine available, especially in low- and middle-income countries, because of narrow production capacity and logistics [2,8,9]. There is an urgent need to optimize the allocation of scarce vaccines.

The optimal allocation depends on the objective of infection control. If the objective is to minimize hospitalizations, it might be best to target those with the highest risk of severe illness upon infection. If the objective is to reduce transmission of infection, it might be best to target the individuals who contribute most to future infections. Similar allocation problems were previously explored for influenza vaccination programmes [10–12]. The allocation of COVID-19 vaccines has been evaluated in combination with NPIs [13–15], with age-varying vaccine efficacy [16], and with different sizes of the vaccine stockpile [17,18]. These studies examined plausible scenarios with numerous combinations of models and parameters; however, the challenge here is that there are innumerable possible allocation schemes to compare.

Here we show a data-driven approach to find optimal allocation schemes, by age group and vaccine type, that minimize either new infections, hospitalizations, or deaths. As per previous studies [13–18], we stratify the population by age, because age is shown to be an important risk factor for susceptibility [19,20], severe illness [21,22], and mortality [21,23,24]. We apply the proposed approach to a simulated epidemic to evaluate its performance. We also test it with the available data on COVID-19 in the Netherlands as of October 2020, when vaccination programme was planned, in order to find optimal allocation schemes for different types of vaccines.

## Results

### Impact of a single unit of vaccination

We are interested in prioritizing a subgroup, to target vaccination of individuals in group *i*, by considering within- and between-subgroup transmissions. To find optimal allocation schemes, the proposed approach relies on establishing the impact of a single unit of vaccine (i.e., the number of doses to fully immunize a single individual), as described in the following three steps.

First, we write an age-stratified transmission process in matrix form by introducing the next generation matrix ***K*** [25–27]. ***K*** gives the number of new infections in a successive generation, such that the number of new infections at time *t*+1 after one generation of infections is ***x***(*t*+1) = ***Kx***(*t*). Note that ***K*** is a *m*×*m* matrix, and we have *m* age groups. We start with a *m*×1 vector of age-specific infection at time *t*, ***x***(*t*).

Second, we define the “impact” of a single unit of vaccination as the reduction in the number of new infections generated by an infected individual. A decrease in the number of infected individuals at time *t*+1, ***x***(*t*+1), is expressed as a result of changes in ***K*** and in the number of infected individuals ***x***(*t*) due to vaccinating one individual. With simplified notation, we can write this as ***x***′(*t*+1) = ***K***′***x***(*t*)+***Kx***′(*t*), where ***K***′ and ***x***′(*t*) are derivatives with respect to the number of vaccinated individuals; ***K***′***x***(*t*) is the direct effect of vaccinating an individual by removing them from the susceptible population and ***Kx***′(*t*) is the indirect effect of vaccinating an individual by reducing onward infections (see Eq S4 and Eq S7 for full notation).

Third, the main interest here is to approximate ***K*** using observed epidemiological data. By approximating ***K***, we can calculate above-defined changes without knowing the detailed contact information between groups. To derive the approximated form, we require that at-risk contacts are reciprocal. With this condition, ***K*** can be safely approximated by the combination of the force of infection $\frac{x_{i}(t)}{s_{i}(t)}$ (i.e., incidence rate of new infections *x*_*i*_(*t*) per susceptible individual *s*_*i*_(*t*)) and the incidence rate of new infections per individual $\frac{x_{i}(t)}{n_{i}}$, and its approximation error is guaranteed to be small if the observation interval for new infections is more than two generation intervals [28] (see detailed derivation in **S1 Text**).

Using the above results, when age group *i* is targeted for vaccination, its impact can be measured as the contribution of the change in group *i* to the relative reduction in the number of new infections after one generation of infection (see Eq S11 in **S1 Text**). As a result of the approximation of the next generation matrix ***K***, we can define this quantity as the “importance weight” of infection $y_{i}^{\left(I\right)}$ only with group-specific inputs, given by

$$y_{i}^{\left(I\right)}=Rfg\left(q_{i}^{\left(S\right)}+q_{i}^{\left(T\right)}\right)\frac{c_{i}}{a_{i}}\frac{x_{i}(t)}{s_{i}(t)}\frac{x_{i}(t)}{n_{i}}$$ (1)

where *R* is the reproduction number, *f* and *g* are normalizing factors, $q_{i}^{\left(S\right)}$ and $q_{i}^{\left(T\right)}$ are vaccine efficacies for susceptibility and transmissibility in age group *i*, *c*_*i*_ is per contact probability of transmitting infection for age group *i*, and *a*_*i*_ is per contact probability of acquiring infection for age group *i*. We can interpret the quantity $y_{i}^{\left(I\right)}$ as the expected reduction in the number of new infections generated by an infected individual after introducing a single unit of vaccine in group *i*, compared with the counterfactual situation where no vaccine is introduced.

The importance weight can be generalized for other disease outcomes such as quality-adjusted life year (QALY) and disability-adjusted life year (DALY). We find that the generalized form of *Eq*.1 for other disease outcomes can be written as the product of the relative change in the number of new infections $y_{i}^{\left(I\right)}$ and a disease progression rate (see the derivation in **S1 Text**). To illustrate its application, we introduce the importance weight of hospitalization $y_{i}^{\left(H\right)}$ and death $y_{i}^{\left(D\right)}$, which are defined as the relative reduction in the number of hospitalizations and deaths;

$$y_{i}^{\left(H\right)}=\eta_{i}y_{i}^{\left(I\right)}$$ (2)

and

$$y_{i}^{\left(D\right)}=\mu_{i}y_{i}^{\left(I\right)}$$ (3)

where *η*_*i*_ is the infection hospitalization rate and *μ*_*i*_ is the infection mortality rate for age group *i*.

### Prioritization algorithm

Given a limited stockpile of vaccines, we assess the expected impacts of a single vaccination on the number of new infections, hospitalization, or deaths, with importance weights (i.e., $y_{i}^{\left(I\right)},\;y_{i}^{\left(H\right)}$ and $y_{i}^{\left(D\right)}$ shown in Eqs 1-3). In the case that there are multiple types of vaccines, we can define importance weights by vaccine type. To illustrate the algorithm proposed in this study, we use the example of minimization of hospitalization, letting $y_{i}^{\left(H)(j\right)}$ denote the importance weight of hospitalization (H) for vaccine type *j* in age group *i*. By comparing age and vaccine type specific importance weights, the sequential allocation is performed as described below:

Step-1: Decide the objective of infection control (in this example, minimizing hospitalization (H))
Step-2: Calculate importance weights $y_{i}^{\left(H)(j\right)}$ per age-group *i* and vaccine type *j*
Step-3: Find a combination of age-group *i* and vaccine type *j* that has the largest importance weight; this provides the selected age group and selected vaccine type.
Step-4: Allocate a single unit of the selected vaccine to the selected age-group
Step-5: Re-calculate importance weights by decreasing the weights in the targeted age-group, as $y_{i}^{\left(H)(j\right)}+\frac{dy_{i}^{\left(H)(j\right)}}{du_{i}}$. Others remain the same.
Step-6: Repeat above until the end of vaccine stockpile.

Note that in step-5 all the importance weights of the age group *i* are updated. This is because the allocation of one vaccine type depletes susceptible and infectious individuals in the targeted age group, and thus it affects the expected impacts of other vaccines from next iterations (see detailed derivation in **S1 Text**). The pseudo code for this algorithm is provided in **S2 Table**. Here, the intention is to present the algorithm in a straightforward manner; improvements to reduce the runtime are possible.

There are four conditions that should be met; (i) the epidemic grows exponentially over the time interval, (ii) at-risk contacts are reciprocal, (iii) the observation interval for new infections is sufficiently long, and (iv) there is no major change in the age distribution of the risk of infection. With these assumptions, we can reconstruct the (approximated) next generation matrix and calculate the expected impact on each outcome due to vaccination, without detailed information about contacts between groups [28].

### Test against simulated data

**Fig 1:**
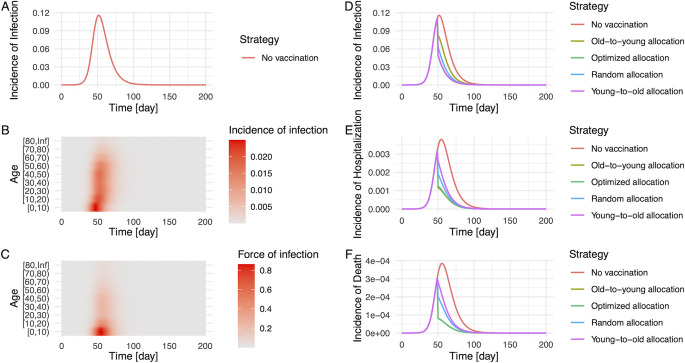
Simulated epidemic and evaluation of the impact of vaccination by allocation strategy. The epidemic is simulated by an age-structured SIR model. *R*_0_ and generation time were set as 1.2 and 5 days, respectively. The population was stratified by 10-year age bin, and a contact matrix of the Netherlands in June 2020 was used for the simulation [32]. Panel **(A)** illustrates the total incidence of infection in the population, and age-specific incidences **(B)** and the force of infection **(C)** reflect heterogeneous contacts between age-groups. The impact of vaccination on the number of infections **(D)**, hospitalizations **(E)**, and deaths **(F)** was compared under five different strategies; no vaccination (red), allocation from old to young groups (yellow), young to old groups (purple), at random (blue), and optimized allocation (green). In panel **(D)**, curves of the optimized allocation and the young-to-old allocation are overlapped. In panel **(E)** and **(F)**, curves of the optimized allocation and the old-to-young allocation are overlapped. For simplicity, the vaccination coverage was set as 40%, and the effect of vaccines was in place at day 50 (from the initial time point of the simulation), resulting in the immediate depletion of susceptible and infected individuals on that day.

We test the performance of the proposed algorithm using a simulated epidemic. **Fig 1A** illustrates the generated epidemic curve where we set the basic reproduction number *R*_0_ to 1.2 and the generation time as 5 days, based on the estimates of SARS-CoV-2 infections, following previous modelling studies [13,16] (see **Materials and Methods** for details of simulation settings). Although only partial observations on the incidence and force of infection are used as inputs, the allocation strategies yielded by our algorithm perform better than other strategies that we tested in most cases (i.e., random allocation, allocation from young to old groups, allocation from old to young groups, and no vaccination) (**Fig 1D, 1E and 1F**).

### Age distribution of allocated vaccines by prioritization scheme

**Fig 2:**
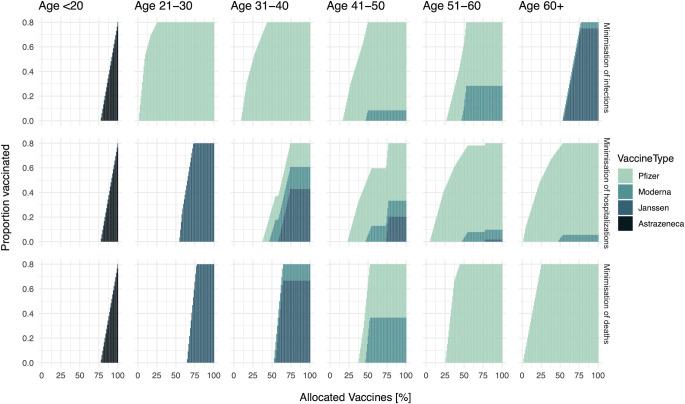
The order of vaccine allocation by age and by prioritization strategy for a stockpile that suffices to vaccinate 80% of the population. From the top row, the objective is the minimization of infections, hospitalizations, and deaths respectively. From the left column, the proportion of vaccinated among age <20, 21–30, 31–40, 41–50, 51–60, 60+ are plotted over allocated vaccines. Note that the X-axis shows the percentage of allocated vaccines.

We apply the proposed approach to epidemiological data on COVID-19 in the Netherlands as of October 2020. We allocate a vaccine stockpile that covers 80% of the total population. The breakdown of the stock is Pfizer (46%), AstraZeneca (22%), Moderna (8%), and Janssen (24%), following the logistics plan before the vaccination programme. Higher efficacious vaccines are allocated first, and then lower efficacious vaccines are distributed later on (**Figs 2 and S2**). **Fig 2** shows the detailed breakdown of allocated vaccines by age group and vaccine type in each allocation scheme, and all the schemes start with the highest efficacious vaccine (i.e., Pfizer vaccine). Since high vaccine efficacy results in larger impacts per vaccination (Eq 1), it is natural to prioritize the allocation of higher efficacious vaccines.

Depending on the objective of infection control, the type of vaccines that each age group receives would differ. If a specific age group is significantly contributing to the objective, it is better to distribute higher efficacious vaccines to that group. For example, there is a large contribution of age 21–30 for the number of infections (**S1 Fig**), and thus higher efficacious vaccines are distributed to that group if the objective is to minimize the number of infections (top row in **Fig 2**). If we wish to minimize the number of hospitalizations or deaths, those vaccines would be distributed to the elderly (second and third rows in **Fig 2**).

The optimal timing of switching from one age group to another also varies by objective. When we set the objective as the minimization of the number of infections or hospitalizations, the selected allocation orders for these two objectives suggest to distribute vaccines to several age-groups in parallel (first and second rows in **Figs 2 and **S3). By contrast, when we set the objective as the minimization of the number of deaths, the allocation scheme generally focused on one age group, from old to young, and did not switch to the next age group until the vaccination of the first age group (i.e., age 60+) is finished (third row in **Figs 2 and **S3). In terms of the order and the switching timing, the selected allocation scheme that minimizes deaths is concordant with the current allocation policy in the Netherlands [29].

### Different benefits between vaccine prioritization strategies

**Fig 3:**
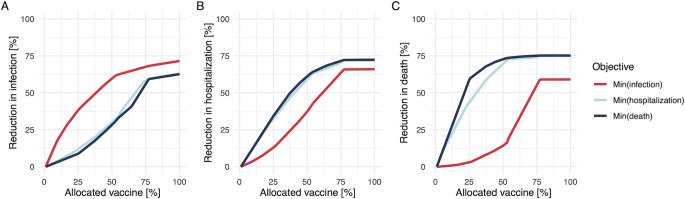
Performance of allocation schemes on different objectives for a stockpile that suffices to vaccinate 80% of the population. The breakdown of the stock is Pfizer (46%), AstraZeneca (22%), Moderna (8%), and Janssen (24%). The Y-axis shows the percentage reduction in the number of infections (A), hospitalizations (B), and deaths (C), and the X-axis is the percentage of allocated vaccines. Red, light blue, and dark blue plots indicate the allocation strategies to minimize the number of infections, hospitalizations, and deaths respectively. The starting point of effective reproduction number (i.e., the reference point without any vaccination) was set as 1.2.

Allocation schemes that are optimized for one objective may not be optimal with respect to another, as illustrated by our simulations. If we choose to minimize the number of infections, that allocation scheme is not efficient for the minimization of deaths (**Fig 3A**). In contrast, if we wish to minimize the number of hospitalizations or deaths (**Fig 3B and 3C**), those strategies are not efficient for minimizing infections. Especially, the difference in the expected reduction is larger at the early phase of allocations; this is because mainly younger age groups are drivers of transmission (**S1A Fig**), while younger individuals are not in high-risk groups in terms of hospitalization or death (**S1F and S1G Fig**).

The proposed algorithm finds the best solution at each allocation step. This results in an optimal solution for small stockpiles, but this local optimal solution is not necessarily optimal for larger stockpiles (so called “greedy algorithm” [30]). To elucidate this property, we simulate an alternative situation, before the approval of the Janssen vaccine, where the breakdown of the stock is Pfizer (40%), AstraZeneca (40%), and Moderna (20%). **S4 Fig** illustrates that the allocation scheme to minimize infections results in nearly equal reduction of infections at the end of allocations compared to the other two schemes, although it performed best at the beginning phase.

## Discussion

The present study proposes a prioritization algorithm that can find an optimal allocation of vaccines to different age groups, even with a limited amount of data. Our simulation results show how optimal allocation differs depending on the objective. We apply the algorithm to available Dutch epidemiological data on COVID-19, and the allocation scheme that minimizes deaths is concordant with the current policy in the Netherlands that allocates vaccines from old to young, given an epidemiological situation with ongoing transmission [29].

The proposed method provides first principles to find optimal allocation schemes with limited data, and the output can also be used as a complementary tool to existing computational approaches. Previous studies hinged on dynamic modelling to determine the prioritization of vaccine allocation [13,16,17], and our algorithm can inform a near-optimal distribution of vaccines as input values for those simulations. The proposed method can be used as a cross-check of assumptions in dynamic models, because it does not require the detailed information on contact matrices or non-pharmaceutical interventions. In the COVID-19 pandemic, we have already observed immediate changes of the age distribution of reported cases [20,31], and contact patterns during lockdown are different from usual patterns [32]. The strength of our approach is that it relies only on routine surveillance data and captures changes in contact patterns through those data.

Choosing a different objective for COVID-19 control implies choosing a different optimal allocation scheme. In the case of SARS-CoV-2 infection, individuals who are at higher risk of severe illness and who transmit are different [19,22]. Our results (**Figs 1 and 3**) illustrated that, if we weigh an objective (e.g., minimization of infections) and choose a strategy, the selected scheme is not necessarily efficient for the other objectives (e.g., minimization of hospitalizations and deaths). This implication is consistent with other dynamic modelling approaches that suggest the elderly should be prioritized to minimize the number of deaths [17,18]. In our analysis, the difference in the reduction of each outcome was larger at the earlier phase of vaccination (**Fig 3**), indicating the importance of decision-making in the beginning stage of allocations. While vaccine rollout has progressed rapidly in the first half of 2021 in high-income countries, there is large vaccine inequity globally [33]. In many low- and middle-income countries vaccine rollout is hindered by limited supply. An algorithm, such as the one presented here, can be very useful to prioritize vaccine allocation in those countries where maximum impact on disease outcomes must be achieved by a small supply of vaccines. Besides, the proposed method can be easily generalized for a wider range of objectives, by multiplying a disease progression rate (**S1 Text**). The contribution of this study is to provide a solution how to determine the subgroup with the largest contribution to different outcomes, given limited data.

When the proposed algorithm is applied, several assumptions and underlying conditions of input values should be checked. First, confirmed case counts may not reflect the actual infection dynamics in the population, depending on the level of ascertainment [34,35]. Our approach relies on the estimates of group-specific incidence and force of infection, as the best proxy of ongoing transmission, and thus potential biases in the surveillance should be carefully scrutinized. Comparing group-specific case reports to serological evidence for infections in the groups may help to identify differences in under-reporting. Second, our modelling simplified offering vaccine doses as a single event and parameterized vaccine efficacies as the ability of reducing infections (***Q***_***S***_) and blocking transmissions (***Q***_***T***_), separately. While there is an advantage to be able to evaluate various characteristics of vaccines by incorporating both the marginal benefit and direct protection, additional supportive evidence on the vaccine efficacy is required. Third, we assume that risk contacts are reciprocal and that individuals are randomly mixing in each group. Although the reciprocity is not violated by a broad class of diseases [32,36], if there were a specific age group that refuses vaccination, and if its proportion became significantly large, that kind of clustering effect might influence the result of approximation of transmission processes.

In conclusion, the present study proposes an approach to find an optimal allocation of vaccines for various objectives, given routine surveillance data. The principle of allocation is simple and interpretable. These features are essential for decision making and for answering to ethical questions that are inherent to allocation of scarce resources. In the context of COVID-19 control, the ability to base important decisions on real-time data, rather than the assumed effect of contact patterns and non-pharmaceutical interventions, might provide a more robust scientific basis for COVID-19 control.

## Materials and methods

### Covid-19 epidemic data in the Netherlands

In the application to the COVID-19 data in the Netherlands, we aimed to perform the proposed algorithm with available data as of October 2020. Our objective here was to illustrate the best strategy at that time.

The population data was stratified into six age groups [<20, 21–30, 31–40, 41–50, 51–60, 60+]. For each age group, we used data on the population size, seroprevalence, incidence of notified cases, maximum vaccine uptake (i.e., willingness to be vaccinated), COVID-19 hospitalization rate, COVID-19 mortality rate, and vaccine efficacy against infection and transmission (**S1 Fig**). As of October 2020, the latest seroprevalence data was obtained from the Pienter-Corona study among a representative sample of the Dutch population, collected in June 2020 [37]. We used this data to calculate the proportion of susceptible individuals per group, that is, 1 –seropositive rate. Note that we used the incidence of notified cases directly as input data, without adjusting reporting rates by age, as there was no targeted testing policy during that period.

In addition to the Dutch data described above, we used infection hospitalization rate and infection mortality rate that were estimated by published studies based on pooled analyses over 45 countries [22,24]. The maximum vaccine uptake was assumed to be 80% for all age groups, following previous modelling studies [13,16]. We assumed the same vaccine efficacies against infection and transmission, which are constant over age groups, based on literatures [38–41]. Epidemiological data in the Netherlands and other input data are visualized in **S1 Fig**. To calculate the expected decrease in the number of new infections, hospitalizations, and deaths, as a function of the number of allocated vaccines, the starting point of effective reproduction number *R* (i.e., the reference point without any vaccination) was set to 1.2 based on the estimates in the Netherlands during October 2020 [42].

We allocated a vaccine stockpile that covers 80% of the total population. The breakdown of the stock is Pfizer (46%), AstraZeneca (22%), Moderna (8%), and Janssen (24%). Note that we considered the unit of vaccines as a set of full doses; for example, the Pfizer vaccine needs to be administered twice, and the set of those two doses was defined as a single unit here. We assumed that one person can receive only one type of vaccines. Thus, 80% of the population was vaccinated when all vaccines were allocated.

We can apply the proposed algorithm adaptively for smaller stockpiles, update observations, apply the algorithm again, and so on; for example, if it takes 14 days to allocate vaccines to 10% of the population, we will be able to obtain new observed data after 14 days, and subsequently, the input can be updated for the algorithm to simulate the next batch.

### Performance evaluation with simulated epidemics

We simulated an epidemic, using a deterministic SIR model, where all parameters were known a priori. We evaluated five different allocation strategies: optimal allocation for each objective (i.e., minimization of infections, hospitalizations, and deaths) determined by the proposed algorithm; random allocation; allocation from young to old groups; allocation from old to young groups; and no vaccination. To quantify the impact of vaccination in each strategy, we took the “no vaccination” scenario as a natural reference point. The population was stratified by 10 year age group, since a contact matrix of the Netherlands in June 2020 was available with those age bins and used for the simulation [32]. An age-structured SIR model was used to generate an epidemic curve where *R*_0_ was set as 1.2 with the fixed generation time as 5 days, based on the estimates of SARS-CoV-2 infections following previous modelling studies [13,16]. For simplicity, per contact probability of acquiring infection (*a*_*i*_) and per contact of transmitting infection (*c*_*i*_) were assumed to be equal (that is, *a*_*i*_ = *c*_*i*_ for all *i*), and the vaccine efficacy was 0.946 based on the estimate for Pfizer [38]. The available vaccine stock was set as 40% coverage of the population, which covers a half of the population that are willing to get vaccinated.

As a practical application, observable information (i.e., the incidence of infection and the force of infection) until day 45 was used as inputs, where day 0 is the initial time point of a simulated SIR epidemic. The optimal distribution of vaccines to each age group was yielded by the proposed algorithm. Note that the algorithm does not use the contact matrix. In each scenario, the effect of allocated vaccines became in place at day 50 all at once, resulting in the immediate depletion of susceptible and infected individuals in the population. We generated hypothetical epidemics with the immediate mass vaccination scenario to visualize the impacts of different allocation strategies, but of course other strategies, such as repeated allocation of smaller stockpiles, are also possible. Replication code and data are available on GitHub (https://github.com/fmiura/VacAllo_2021).

### Derivation of importance weights

For a broad class of compartmental models, the disease transmission is described as transitions from discrete states (e.g., susceptible-infectious-recovered states in the SIR model), and the dynamics is generated by a system of nonlinear ordinary differential equations (ODEs) that depicts the change over time. By linearizing ODEs, any (linear) system can be described by a matrix form [26]. Within this linearized subsystem, one can determine the reproduction number *R* as the dominant eigenvalue of the next generation matrix ***K*** [25–27].

The first step is to relate the observed data to ***K***. If at-risk contacts are reciprocal, ***K*** becomes a product of symmetric matrices and diagonalizable. This condition allows the decomposition of ***K***, and thus we can approximate ***K*** by the top left and right eigenvectors that can be (approximately) described by the incidence of new infections and force of infection [28].

Once the matrices are specified, we can evaluate the impact of a single unit of vaccination, as the sensitivity (or elasticity) of the transition matrix (see the general idea of the sensitivity of a matrix in [43], and its application in infectious disease epidemiology in [27,44]). The change in the number of infections per single vaccination can be formulated as the result of depletion of susceptible and infectious individuals from the population (Eq S4 in **S1 Text**), and subsequently, we obtain its effect on the dominant eigenvalue of the next generation matrix that was already introduced in the first step as an approximation with observed data. The expected impact here is defined as the importance weight; if we allocate a single unit of vaccine to the group with the largest importance weight, that results in the minimization of the dominant eigenvalue, that is, the expected number of infections, hospitalizations, or deaths in total.

## Supporting information

S1 Fig Age-specific input data. Age-specific input data for the proposed algorithm to obtain optimal allocation schemes. (A) Population structure in the Netherlands in 2019 (B) Seroprevalence observed in the Pienter-Corona study among a representative sample of the Dutch population in June 2020 [1]. (C) Incidence of notified cases, in 30 days before October 19, 2020 (D) Vaccine Efficacy by vaccine type. From lighter to darker blue, bars indicate Pfizer Moderna, Janssen, AstraZeneca. Note that the constant efficacy by age here is an assumption, based on reported over all vaccine efficacies [2–5]. (E) Maximum vaccine uptake per age group. 80% for all groups is assumed here. (F) COVID-19 hospitalization rate. These values are based on [6]. (G) COVID-19 mortality rate. These values are based on [7]. Black bars indicate Dutch specific data (i.e., (A), (B), and (C)), while other colored bars indicate data from literature (i.e., (D), (E), (F), and (G)).
(TIFF)

S2 Fig Simulated vaccine allocations by age and by vaccine type. Vaccine allocations based on simulated data when the objective is to minimize the number of infections ((A) and (B)), hospitalizations ((C) and (D)), and deaths ((E) and (F)). In left three panels, from lighter to darker blue, bars indicate Pfizer Moderna, Janssen, AstraZeneca. In right three panels, the darker color shows the older age groups, and age bins are [20<,21–30,31–40,41–50,51–60,60+].
(TIFF)

S3 Fig Simulated prioritization of age-group by allocation scheme. Vaccine allocation based on simulated data when the objective is to minimize the number of infections (A), hospitalizations (B), and deaths (C). The darker color shows the older age groups, and age bins are [20<,21–30,31–40,41–50,51–60,60+].
(TIFF)

S4 Fig Simulated impact of vaccination. Performance of allocation schemes on different objectives for a stockpile that suffices to vaccinate 80% of the population. The breakdown of the stock is Pfizer (40%), AstraZeneca (40%), and Moderna (20%). The Y-axis shows the percentage reduction in the number of infections (A), hospitalizations (B), and deaths (C), and the X-axis is the percentage of allocated vaccines. Red, light blue, and dark blue plots indicate the allocation strategies to minimize the number of infections, hospitalizations, and deaths respectively. The starting point of effective reproduction number (i.e., the reference point without any vaccination) was set as 1.2.
(TIFF)

S1 Table Notation and meaning of variables. (DOCX)

S2 Table Pseudo code of the allocation algorithm. (DOCX)

S1 Text Mathematical details. (DOCX)
