# Supplementary figures and images for: Optimal vaccine allocation for COVID-19 in the Netherlands: A data-driven prioritization
Source: PLoS Comput Biol. 2021 Dec 13;17(12):e1009697. doi: 10.1371/journal.pcbi.1009697 (PMC8699630; doi:10.1371/journal.pcbi.1009697)

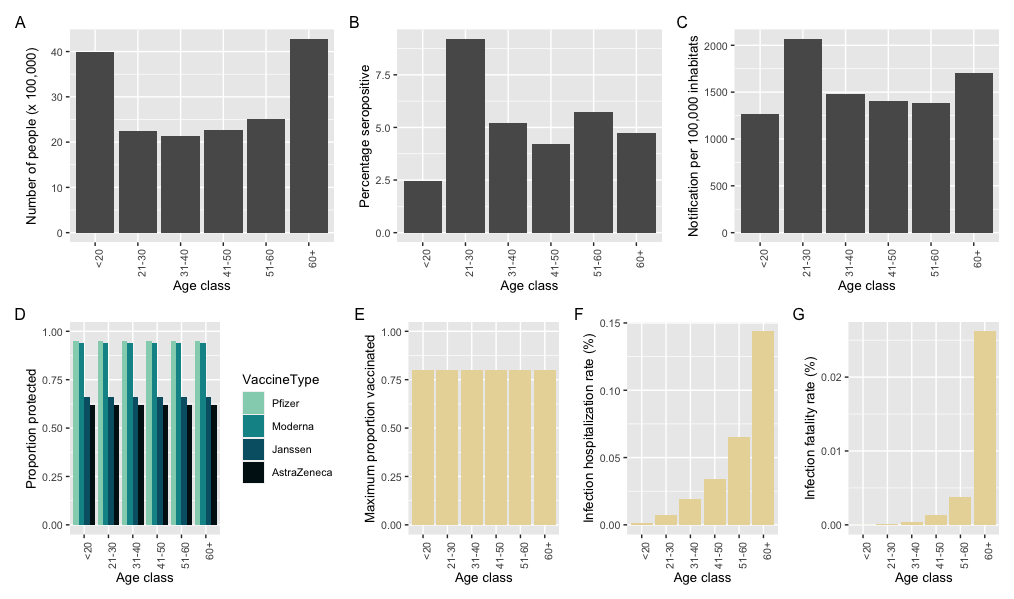

Supplement: S1 Fig — Age-specific input data for the proposed algorithm to obtain optimal allocation schemes. (A) Population structure in the Netherlands in 2019 (B) Seroprevalence observed in the Pienter-Corona study among a representative sample of the Dutch population in June 2020 [1]. (C) Incidence of notified cases, in 30 days before October 19, 2020 (D) Vaccine Efficacy by vaccine type. From lighter to darker blue, bars indicate Pfizer Moderna, Janssen, AstraZeneca. Note that the constant efficacy by age here is an assumption, based on reported over all vaccine efficacies [2–5]. (E) Maximum vaccine uptake per age group. 80% for all groups is assumed here. (F) COVID-19 hospitalization rate. These values are based on [6]. (G) COVID-19 mortality rate. These values are based on [7]. Black bars indicate Dutch specific data (i.e., (A), (B), and (C)), while other colored bars indicate data from literature (i.e., (D), (E), (F), and (G)). (TIFF) [file pcbi.1009697.s001.tiff]

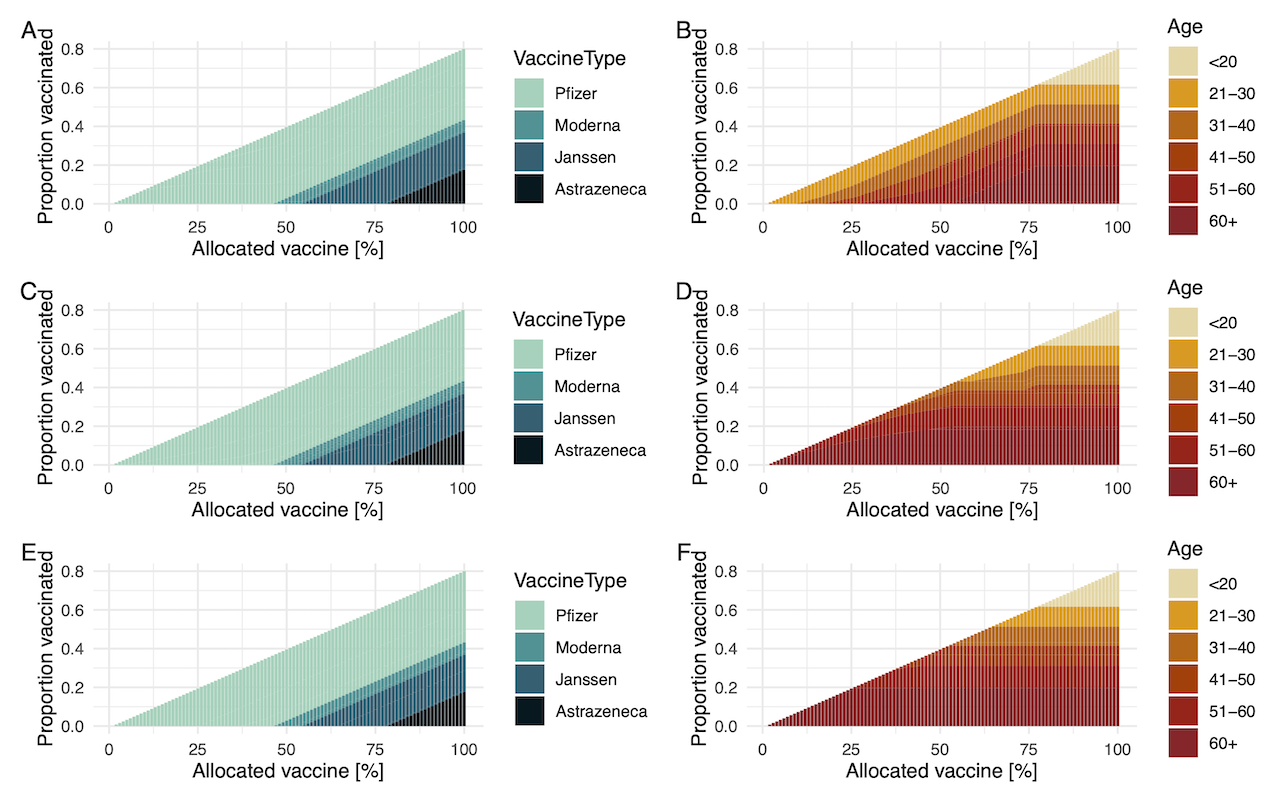

Supplement: S2 Fig — Vaccine allocations based on simulated data when the objective is to minimize the number of infections ((A) and (B)), hospitalizations ((C) and (D)), and deaths ((E) and (F)). In left three panels, from lighter to darker blue, bars indicate Pfizer Moderna, Janssen, AstraZeneca. In right three panels, the darker color shows the older age groups, and age bins are [20<,21–30,31–40,41–50,51–60,60+]. (TIFF) [file pcbi.1009697.s002.tiff]

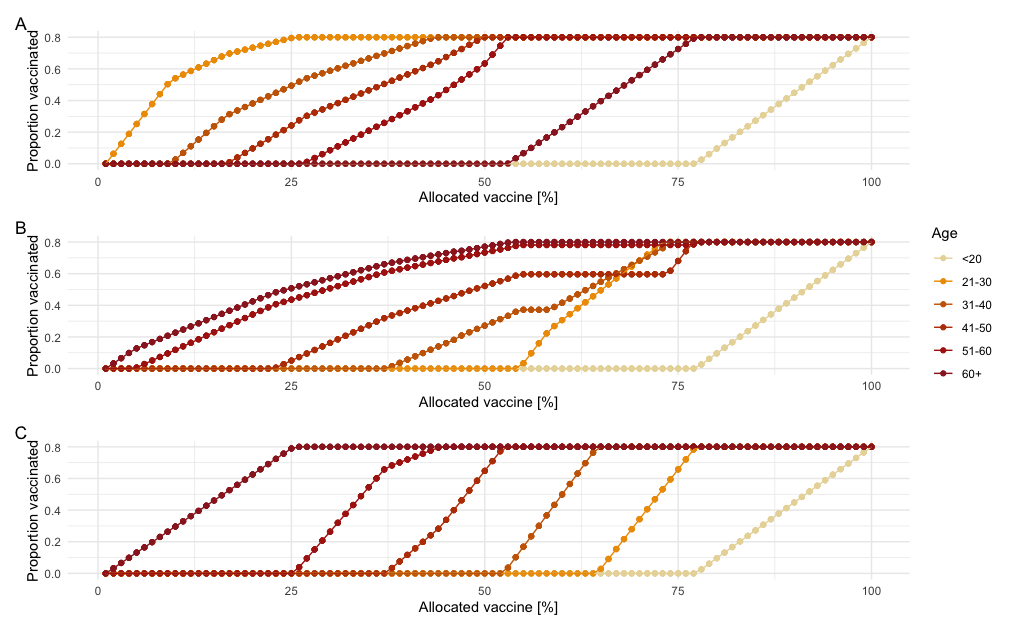

Supplement: S3 Fig — Vaccine allocation based on simulated data when the objective is to minimize the number of infections (A), hospitalizations (B), and deaths (C). The darker color shows the older age groups, and age bins are [20<,21–30,31–40,41–50,51–60,60+]. (TIFF) [file pcbi.1009697.s003.tiff]

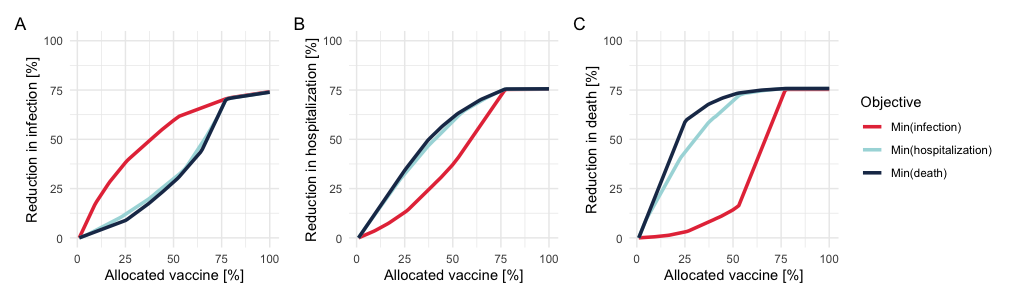

Supplement: S4 Fig — Performance of allocation schemes on different objectives for a stockpile that suffices to vaccinate 80% of the population. The breakdown of the stock is Pfizer (40%), AstraZeneca (40%), and Moderna (20%). The Y-axis shows the percentage reduction in the number of infections (A), hospitalizations (B), and deaths (C), and the X-axis is the percentage of allocated vaccines. Red, light blue, and dark blue plots indicate the allocation strategies to minimize the number of infections, hospitalizations, and deaths respectively. The starting point of effective reproduction number (i.e., the reference point without any vaccination) was set as 1.2. (TIFF) [file pcbi.1009697.s004.tiff]
