# Supplementary material for: Optimal vaccine allocation for COVID-19 in the Netherlands: A data-driven prioritization
Source: PLoS Comput Biol. 2021 Dec 13;17(12):e1009697. doi: 10.1371/journal.pcbi.1009697 (PMC8699630; doi:10.1371/journal.pcbi.1009697)
Supplement: S1 Table — (DOCX) [file pcbi.1009697.s005.docx]

**S1 Table**. Notation and meaning of variables

| Symbol | Meaning |
| --- | --- |
| $\boldsymbol{K}$ | Next generation matrix with elements $k_{ij}$ |
| $\boldsymbol{S}$ | Diagonal matrix with group-specific number of susceptible individuals $s_{i}\boldsymbol{(}t\boldsymbol{)}$ in group *i* at time *t* |
| $\boldsymbol{A}$ | Diagonal matrix with per contact probability of acquiring infection $a_{i}$ for group *i* |
| $\boldsymbol{B}$ | Matrix with group-specific contact parameter $b_{ij}$ (i.e., the proportion of group *i* contacted by an infective in group *j*) |
| $\boldsymbol{C}$ | Diagonal matrix with per contact probability of transmitting infection $c_{i}$ for group *i* |
| $\boldsymbol{N}$ | Diagonal matrix with the population size $n_{i}$ of group *i* |
| $\boldsymbol{D}$ | Diagonal matrix with group-specific infection mortality rate $\mu_{i}$ on the diagonal |
| $\boldsymbol{H}$ | Diagonal matrix with group-specific infection hospitalization rate $\eta_{i}$ on the diagonal |
| $\boldsymbol{U}$ | Diagonal matrix with the number of vaccines $u_{i}$ given to group *i* on the diagonal |
| $\boldsymbol{P}^{\left( I \right)}$ | Projection matrix that describes expected reductions in new infections in group *i* with the dominant eigenvalue ${\lambda_{1}}^{\left( I \right)}$ |
| $\boldsymbol{Q}_{\boldsymbol{S}}$ | Diagonal matrix with vaccine efficacy against acquiring infection $q_{\boldsymbol{i}}^{\boldsymbol{(}S\boldsymbol{)}}$on the diagonal |
| $\boldsymbol{Q}_{\boldsymbol{T}}$ | Diagonal matrix with vaccine efficacy against transmission $q_{\boldsymbol{i}}^{\boldsymbol{(}T\boldsymbol{)}}$on the diagonal |
| $\boldsymbol{u}$ | Vector with the number of vaccinated individuals $u_{i}$ in group *i* |
| $\boldsymbol{x(t)}$ | Vector with group-specific number of new infections $x_{i}\boldsymbol{(}t\boldsymbol{)}$ in group *i* at time *t* |
| $\boldsymbol{h(t)}$ | Vector with group-specific number of new infections $h_{i}\boldsymbol{(}t\boldsymbol{)}$ in group *i* at time *t* |
| f,g | Normalization factors |
| $R$ | Reproduction number (top eigenvalue of the next generation matrix $\boldsymbol{K}$) |
| $\tau$ | Generation interval of infections |
| $z^{\boldsymbol{(j)}}$ | Number of available vaccine stocks for type *j* |
