# Supplementary material for: Optimal vaccine allocation for COVID-19 in the Netherlands: A data-driven prioritization
Source: PLoS Comput Biol. 2021 Dec 13;17(12):e1009697. doi: 10.1371/journal.pcbi.1009697 (PMC8699630; doi:10.1371/journal.pcbi.1009697)
Supplement: S2 Table — (DOCX) [file pcbi.1009697.s006.docx]

**S2 Table**. Pseudo code of the allocation algorithm

| **Algorithm 1** |
| --- |
| Input variables:   - Number of available vaccine stocks $z^{(j)}$ for each vaccine type *j* - Group-specific probability of acquiring infection per contact $a_{i}$ and of transmitting infection per contact $c_{i}$ - Group-specific population size $n_{i}$ - Group-specific number of new infections $x_{i}\boldsymbol{(}t\boldsymbol{)}$ in group *i* at time *t* - Group-specific number of susceptible individuals $s_{i}\boldsymbol{(}t\boldsymbol{)}$ in group *i* at time *t* - Group- and vaccine type-specific vaccine efficacy against acquiring infection $q_{i}^{(S)(j)}$ - Group- and vaccine type-specific vaccine efficacy against transmission $q_{i}^{(T)(j)}$ - Initial value of the effective reproduction number *R* |
| Pseudo code:   - Define the objective of infection control (e.g., the number of hospitalizations) - Calculate initial importance weights $y_{i}^{\left( H \right)(j)}$ per age group *i* per vaccine type *j* - Set the number of allocated vaccine type *j* for age group *i* as 0: $u_{i}^{(j)}\leftarrow0$ - Run loops below until (i) all $z^{(j)}$becomes zero OR (ii) all group reach the maximum uptake:   **For** *j* = 1, 2, …, *J* **do:**  **For** *i* = 1, 2, …, *I* **do:**  Find the largest importance weight $y_{i*}^{\left( H \right)(j*)}$  **If** $y_{i*}^{\left( H \right)(j*)}=y_{i}^{\left( H \right)(j)}$ **then:**  Allocate a single unit of vaccine type *j* to the selected group *i*: $u_{i}^{(j)}\leftarrow u_{i}^{(j)}+1$ ; $z^{(j)}\leftarrow z^{(j)}-1$  Update all importance weights of the selected group *i*: $y_{i}^{\left( H \right)(j)}\leftarrow y_{i}^{\left( H \right)(j)}+\frac{dy_{i*}^{\left( H \right)(j*)}}{du}$  **Else:**  Keep the importance weights in the unselected group *i*: $y_{i}^{\left( H \right)(j)}\leftarrow y_{i}^{\left( H \right)(j)}$  **If** $z^{(j)}=0$ **then:**  Update all importance weights of the vaccine type *j* that is out of stock: $y_{i}^{\left( H \right)(j)}\leftarrow0$  **If** $\sum_{j} u_{i}^{(j)}=(maximum vaccine updake of group i)$ **then:**  Update all importance weights of the selected group *i* that reaches the max uptake: $y_{i}^{\left( H \right)(j)}\leftarrow0$  **End** |
| **End** |
| Output variables:   - The number of allocated vaccine type *j* for age group *i* as a function of iteration $l$: $u_{i}^{(j)}(l)$ - Importance weights per age group *i* per vaccine type *j* as a function of iteration $l$: $y_{i}^{\left( H \right)(j)}(l)$ |
