# Supplementary material for: Optimal vaccine allocation for COVID-19 in the Netherlands: A data-driven prioritization
Source: PLoS Comput Biol. 2021 Dec 13;17(12):e1009697. doi: 10.1371/journal.pcbi.1009697 (PMC8699630; doi:10.1371/journal.pcbi.1009697)
Supplement: S1 Text — (DOCX) [file pcbi.1009697.s007.docx]

**S1 Text.** Mathematical details

*1. Mathematical details*

*1.1 Objective*

The aim of following calculations is to formulate the expected impact of targeted vaccination. We firstly present our approach to relate the expected changes in the next generation matrix $\boldsymbol{K}$ to the observed epidemiological data (i.e., the number of new infections per group). We then generalize the argument to quantify the expected impact in the number of hospitalizations and deaths.

The following analysis is known as “perturbation analysis” of a matrix in demography and population ecology [1,2], and we will refer to theorems and proofs from literature. For consistent notations, we follow Magnus and Neudecker (1988) [3]; matrices are denoted by upper case bold symbols (e.g., **A**), and vectors are denoted by lower case bold symbols (**n**). Note that we define the derivatives of a matrix (or vector) as the matrix (or vector) of derivatives of the elements (e.g., $\frac{d\boldsymbol{Y}}{d\boldsymbol{X}}=\left( \frac{{dy}_{ij}}{{dx}_{ij}} \right)$and $\frac{d\boldsymbol{y}}{d\boldsymbol{x}}=\left( \frac{{dy}_{i}}{{dx}_{i}} \right)$). All notations and definitions of variables are shown in **S2 Table**.

*1.2 Next generation matrix*

The host population is subdivided into *m* groups. The next generation matrix $\boldsymbol{K}$ gives the number of new infections in a successive generation, such that the number of new infections at time $t+1$ after $1$ generation of infections is $\boldsymbol{x}(t+1) = \boldsymbol{Kx}(t)$. For a large class of transmission models such as susceptible-infected-recovered model (SIR) model, $\boldsymbol{K}$ can be written as

$$\boldsymbol{K=SABC}$$

where matrices $\boldsymbol{S}$, $\boldsymbol{A}$, $\boldsymbol{B}$, and $\boldsymbol{C}$ have the following epidemiological interpretation: $\boldsymbol{S}$ is a diagonal matrix with group-specific number of susceptible individuals $s_{i}(t)$ on the diagonal, $\boldsymbol{A}$ is a diagonal matrix with per contact probability of acquiring infection $a_{i}$ on the diagonal, $\boldsymbol{B}$ is a contact matrix with elements $b_{ij}$, and $\boldsymbol{C}$ is a diagonal matrix with group-specific per contact probability of transmitting infection $c_{i}$ on the diagonal. Note that only $\boldsymbol{S}$ is time-dependent (and thus $\boldsymbol{K}$ is also time-dependent) in the following analysis. For readability, when it is obvious from the context, we do not write the dependency on time. We require that at-risk contacts are reciprocal, and thus the matrix $\boldsymbol{B}$ is assumed to be symmetric and constant over the observation period. Thus, $\boldsymbol{K}$ is diagonalizable as it is a product of symmetric matrices.

*1.3 Approximation by observed infections*

By diagonalizing $\boldsymbol{K}$, we have

$$\boldsymbol{K=W\Lambda}\boldsymbol{W}^{\boldsymbol{-1}}$$

where $\boldsymbol{\Lambda}$ is a diagonal matrix that has eigenvalues $R$, $\lambda_{2}$, $\lambda_{2}$, ..., $\lambda_{m}$ as its diagonal elements and zeros elsewhere, where $R$ is the dominant eigenvalue and is often referred to as the reproduction number. The matrix $\boldsymbol{W}$ has as columns the right eigenvectors $\boldsymbol{w}_{1}$, $\boldsymbol{w}_{2}$,...,$\boldsymbol{w}_{m}$. The matrix $\boldsymbol{W}^{\boldsymbol{-1}}$ is the inverse of the matrix $\boldsymbol{W}$ that has the left eigenvectors $\mathbf{v}_{1}$, $\mathbf{v}_{2}$,...,$\mathbf{v}_{m}$ as its rows. Both right and left eigenvectors are normalized. We require that an infector introduced in an arbitrary group reproduces a finite number of new infections in every group, ensuring that $\boldsymbol{K}$ is primitive. The Perron-Frobenius Theorem guarantees that $R$*,* $\mathbf{w}_{1}$*,* and $\mathbf{v}_{1}$ are real and non-negative [2,4].

After *τ* generations of infections, the number of new infections at time $t+\tau$ is given by $\boldsymbol{x}(t+\tau)\boldsymbol{=}\boldsymbol{K}^{\tau}\boldsymbol{x}(t)=\boldsymbol{W}\boldsymbol{\Lambda}^{\tau}\boldsymbol{W}^{\boldsymbol{-1}}\boldsymbol{x}(t)$. By using both right and left eigenvectors, we can rewrite the formula as

$$\boldsymbol{x}(t+\tau)=\sum_{i} {\lambda_{i}}^{\tau}\boldsymbol{w}_{i}\mathbf{v}_{i}^{\boldsymbol{T}}\boldsymbol{x}(t).$$

Note that $\boldsymbol{x}\left( t \right)$ is a vector that has the number of new infections in age group *i* as its elements, denoted as *x_i_*(*t*) and that the dominant eigenvalue $\lambda_{1}$ is the reproduction number $R$. If the dominant eigenvalue is strictly greater than other eigenvalues, the first term $R\boldsymbol{w}_{1}\mathbf{v}_{1}^{\boldsymbol{T}}$ will eventually dominate the other terms. This characteristic yields the approximated form

$$\begin{aligned} \bar{\boldsymbol{K}}\boldsymbol{=}R\boldsymbol{w}_{1}\mathbf{v}_{1}^{\boldsymbol{T}}\boldsymbol{.\#}\left( Eq.S1 \right) \end{aligned}$$

Now it is of interest to approximate the top right and left eigenvectors, $\mathbf{w}_{1}$ and $\mathbf{v}_{1}$, by observations. If the observation interval is long enough (typically longer than two generations of infections), we can safely approximate the top right eigenvector $\boldsymbol{w}_{1}$ with the number of new infections $\boldsymbol{x}(t)$ [5]. Thus, we have

$$\begin{aligned} \boldsymbol{w}_{1}\approx f\boldsymbol{x}\left( t \right)\#\left( Eq.S2 \right) \end{aligned}$$

where $f$ is the normalizing factor given by $f=\frac{1}{\sum_{i} x_{i}\left( t \right)}$. This result is also known as the strong ergodic theorem (see ref [6], p.86). Since the contact matrix $\mathbf{B}$ is symmetric and thus $\boldsymbol{K}$ is a product of symmetric matrices, there exists a transformation matrix $\boldsymbol{M}$ that transposes $\boldsymbol{K}$, such that $\boldsymbol{MK}\boldsymbol{M}^{\boldsymbol{-1}}\boldsymbol{=}\boldsymbol{K}^{\boldsymbol{T}}$. With this relationship, we can project the top left eigenvector $\mathbf{v}_{1}$ along the top right eigenvector $\mathbf{w}_{1}$, and subsequently

$$\begin{aligned} \mathbf{v}_{1}\approx\frac{g}{f}\mathbf{C}\mathbf{A}^{\boldsymbol{-1}}\mathbf{S}^{\boldsymbol{-1}}\mathbf{w}_{1}\approx g\mathbf{C}\mathbf{A}^{\boldsymbol{-1}}\mathbf{S}^{\boldsymbol{-1}}\boldsymbol{x}\left( t \right)\#\left( Eq.S3 \right) \end{aligned}$$

where $g$ is the normalization factor given by $g=\frac{\sum_{i} x_{i}\left( t \right)}{\sum_{i} \frac{c_{i}}{a_{i}}\frac{{x_{i}\left( t \right)}^{2}}{s_{i}\left( t \right)}}$. See the detailed derivation in the section 3.4. of supporting info in [5].

*1.4 Sensitivity of the number of new infections to targeted vaccination*

*1.4.1 Changes in the number of new infections due to vaccination*

A decrease in $\boldsymbol{x}(t+1)$ is expressed as a result of changes in $\boldsymbol{K}$ and in the number of infected individuals $\boldsymbol{x}(t)$:

$$\begin{aligned} \frac{d\boldsymbol{x}\left( t+1 \right)}{d\boldsymbol{u}}=\frac{d\boldsymbol{K}}{d\boldsymbol{U}}\boldsymbol{x}\left( t \right)+\boldsymbol{K}\frac{d\boldsymbol{x}\left( t \right)}{d\boldsymbol{u}}\#\left( Eq.S4 \right) \end{aligned}$$

where $\frac{d\boldsymbol{K}}{d\boldsymbol{U}}\boldsymbol{x}\left( t \right)$ is the direct effect of vaccinating an individual and removing them from the susceptible population and $\boldsymbol{K}\frac{d\boldsymbol{x}\left( t \right)}{d\boldsymbol{u}}$ is the indirect effect of vaccinating a single individual by reducing onward infections.

*1.4.2 Perturbation in the next generation matrix* $\boldsymbol{K}$

We focus on the impact of vaccination when vaccines are allocated to the group that can be immune or susceptible. The perturbation of next generation matrix $\frac{d\boldsymbol{K}}{d\boldsymbol{U}}$ is expressed in terms of the change in the number of susceptible individuals $\frac{d\boldsymbol{S}}{d\boldsymbol{U}}$ due to vaccination, such that $\frac{d\boldsymbol{K}}{d\boldsymbol{U}}\boldsymbol{=}\left( \frac{d\boldsymbol{S}}{d\boldsymbol{U}} \right)\boldsymbol{(ABC)}$. We denote the vaccine efficacy on susceptibility as $\boldsymbol{Q}_{\boldsymbol{S}}$, and the depletion of susceptible individuals is written as

$$\frac{d\boldsymbol{S}}{d\boldsymbol{U}}\boldsymbol{=-}\boldsymbol{Q}_{\boldsymbol{S}}\boldsymbol{S}\boldsymbol{N}^{-1}$$

where $\boldsymbol{N}$ is a diagonal matrix that has elements $n_{i}$ of total population in each age group $i$. Since $\frac{d\boldsymbol{K}}{d\boldsymbol{U}}\boldsymbol{=}\left( \frac{d\boldsymbol{S}}{d\boldsymbol{U}} \right)\boldsymbol{(ABC)=}\left( \frac{d\boldsymbol{S}}{d\boldsymbol{U}} \right)\boldsymbol{(}\boldsymbol{S}^{-1}\boldsymbol{K)}$, the perturbation of $\boldsymbol{K}$ is

$$\frac{d\boldsymbol{K}}{d\boldsymbol{U}}\boldsymbol{=(-}\boldsymbol{Q}_{\boldsymbol{S}}\boldsymbol{S}\boldsymbol{N}^{-1}\boldsymbol{)(}\boldsymbol{S}^{-1}\boldsymbol{K)}$$

and, as $\boldsymbol{S}$ and $\boldsymbol{N}$ are diagonal matrices, commutativity yields

$$\begin{aligned} \frac{d\boldsymbol{K}}{d\boldsymbol{U}}=-\boldsymbol{Q}_{\boldsymbol{s}}\boldsymbol{N}^{-1}\boldsymbol{K}\#\left( Eq.S5 \right) \end{aligned}$$

The derivation is the same as the one described in section 3.5. of supporting info in [5]. Note that the vaccine efficacy of susceptibility here (i.e., $\boldsymbol{Q}_{\boldsymbol{s}}$) is defined as the probability of protecting infection per infectious contact (see next section 1.4.3 for another effect of vaccination, which considers the prevention of transmission from an infectious individual).

*1.4.3 Perturbation in the number of infected individuals* $\boldsymbol{x}(t)$

If vaccines are allocated also to infected individuals $\boldsymbol{x}(t)$ at time $t$, the change in the number of infected (infectious) individuals is

$$\begin{aligned} \frac{d\boldsymbol{x}\left( t \right)}{d\boldsymbol{u}}=-\boldsymbol{Q}_{T}\boldsymbol{N}^{-1}\boldsymbol{x}\left( t \right).\#\left( Eq.S6 \right) \end{aligned}$$

where $\boldsymbol{Q}_{T}$ is the vaccine efficacy against the transmissibility.

*1.4.4 Importance weight of infection*

By substituting Eq.S5 and Eq.S6 in Eq.S4, the decrease in the number of new infections after one generation is rewritten as

$$\begin{aligned} \frac{d\boldsymbol{x}\left( t+1 \right)}{d\boldsymbol{u}}=\underset{\text{direct effect}}{\underset{\underbrace{}}{-\boldsymbol{Q}_{S}\boldsymbol{N}^{-1}\boldsymbol{Kx}\left( t \right)}}-\underset{\text{in}\text{direct effect}}{\underset{\underbrace{}}{\boldsymbol{K}\boldsymbol{Q}_{T}\boldsymbol{N}^{-1}\boldsymbol{x}\left( t \right)}}.\#\left( Eq.S7 \right) \end{aligned}$$

The interpretation of the first term is the reduction in the number of new infections because　susceptible individuals were depleted (i.e., direct effect), and that of the second term is the effect preventing onward infections because infectious individuals are depleted (i.e., indirect effect).

Now we can relate this sensitivity $\frac{d\boldsymbol{x}(t+1)}{d\boldsymbol{u}}$ to observations. By approximating the next generation matrix by dominant right and left eigenvectors (i.e., Eq.S1), Eq.S7 is rewritten as

$$\begin{aligned} \frac{d\boldsymbol{x}\left( t+1 \right)}{d\boldsymbol{u}}\approx-\boldsymbol{Q}_{S}\boldsymbol{N}^{-1}\bar{\boldsymbol{K}}\boldsymbol{x}\left( t \right)-\bar{\boldsymbol{K}}\boldsymbol{Q}_{T}\boldsymbol{N}^{-1}\boldsymbol{x}\left( t \right)=-\left( \boldsymbol{Q}_{S}\boldsymbol{N}^{-1}R\boldsymbol{w}_{1}\mathbf{v}_{1}^{\boldsymbol{T}}\boldsymbol{+}R\boldsymbol{w}_{1}\mathbf{v}_{1}^{\boldsymbol{T}}\boldsymbol{Q}_{T}\boldsymbol{N}^{-1} \right)\boldsymbol{x}\left( t \right).\#\left( Eq.S8 \right) \end{aligned}$$

We define the projection matrix as $\boldsymbol{P}^{\left( I \right)}=\boldsymbol{Q}_{S}\boldsymbol{N}^{-1}R\boldsymbol{w}_{1}\mathbf{v}_{1}^{\boldsymbol{T}}\boldsymbol{+}R\boldsymbol{w}_{1}\mathbf{v}_{1}^{\boldsymbol{T}}\boldsymbol{Q}_{T}\boldsymbol{N}^{-1}$ and write ${\lambda_{1}}^{\left( I \right)}$and $\boldsymbol{w}_{1}^{(I)}$for the dominant eigenvalue and corresponding right eigen vector of $\boldsymbol{P}^{(I)}$. When vaccination is targeted at the group *i*, using Eq.S2 and S3, we obtain

$$\begin{aligned} Rw_{1i}v_{i1}\approx Rfg\frac{c_{i}}{a_{i}}\frac{{x_{i}\left( t \right)}^{2}}{s_{i}\left( t \right)}.\#\left( Eq.S9 \right) \end{aligned}$$

We use the same approximation method as section 1.3 for the projection matrix $\boldsymbol{P}^{(I)}$ and its top right eigenvector $\boldsymbol{w}_{1}^{(I)}$. Given the sufficient length of observation intervals, we can safely approximate $\boldsymbol{w}_{1}^{(I)}$ by the number of new infections $\boldsymbol{x}(t)$ such that $\boldsymbol{w}_{1}^{(I)}\approx f\boldsymbol{x}\left( t \right)$ (see ref [6], p.86). Since $\boldsymbol{P}^{(I)}\boldsymbol{w}_{1}^{(I)}={\lambda_{1}}^{\left( I \right)}\boldsymbol{w}_{1}^{(I)}$, the contribution of age group *i* to the dominant eigenvalue ${\lambda_{1}}^{(I)}$ is:

$$\begin{aligned} {\lambda_{1}}^{\left( I \right)}w_{i1}^{(I)}=\boldsymbol{P}_{i}^{(I)}w_{i1}^{(I)}\approx\left( Rfg\left( q_{i}^{\left( S \right)}+q_{i}^{\left( T \right)} \right)\frac{c_{i}}{a_{i}}\frac{x_{i}\left( t \right)}{s_{i}\left( t \right)}\frac{x_{i}\left( t \right)}{n_{i}} \right)w_{i1}^{(I)}.\#\left( Eq.S10 \right) \end{aligned}$$

We can interpret the quantity $Rfg\left( q_{i}^{\left( S \right)}+q_{i}^{\left( T \right)} \right)\frac{c_{i}}{a_{i}}\frac{x_{i}\left( t \right)}{s_{i}\left( t \right)}\frac{x_{i}\left( t \right)}{n_{i}}$ as the expected reduction in the number of new infections generated by a typical infected individual in group *i* after introducing a single unit of vaccine. Thus, we define this expected impact of a single vaccination in group *i* on the dominant eigenvalue ${\lambda_{1}}^{\left( I \right)}$ as the importance weight of infection:

$$\begin{aligned} y_{i}^{\left( I \right)}=Rfg\left( q_{i}^{\left( S \right)}+q_{i}^{\left( T \right)} \right)\frac{c_{i}}{a_{i}}\frac{x_{i}\left( t \right)}{s_{i}\left( t \right)}\frac{x_{i}\left( t \right)}{n_{i}}\#\left( Eq.S11 \right) \end{aligned}$$

*1.5 Sensitivity of the number of hospitalizations to targeted vaccination*

*1.5.1 Changes in the number of hospitalizations due to vaccination*

The number of hospitalized individuals $\boldsymbol{h}(t)$ at time $t$ (i.e., $m\times1$ vector with elements $h_{1}$, $h_{2}$, ..., $h_{m}$) is defined as

$$\boldsymbol{h}(t)\boldsymbol{=Hx}(t)$$

where $\boldsymbol{H}$ is a diagonal matrix with group-specific hospitalization rates $\eta_{1}$, $\eta_{2}$, ..., $\eta_{m}$. Suppose that we wish to predict the number of hospitalizations after one generation of infections. We can write the number of new hospitalizations as

$$\boldsymbol{h}(t+1)\boldsymbol{=Hx}(t+1)=\boldsymbol{HKx}(t).$$

Over the observation interval from $t$ to $t+1$, we assume the group-specific probability of hospitalization is constant.

Here we look at the perturbation of the expected number of hospitalizations $\boldsymbol{h}(t+1)$ due to vaccination. Since the infection hospitalization rate matrix $\boldsymbol{H}$ is constant, our interest is in the perturbation of $\boldsymbol{x}(t+1)$. Therefore, using Eq.S4, the decrease in the number of hospitalizations can be written as

$$\begin{aligned} \frac{d\boldsymbol{h}\left( t+1 \right)}{d\boldsymbol{u}}=\frac{d\boldsymbol{Hx}\left( t+1 \right)}{d\boldsymbol{u}}=\boldsymbol{H}\left( \frac{d\boldsymbol{K}}{d\boldsymbol{U}}\boldsymbol{x}\left( t \right)+\boldsymbol{K}\frac{d\boldsymbol{x}\left( t \right)}{d\boldsymbol{u}} \right).\#\left( Eq.S12 \right) \end{aligned}$$

*1.5.2 Importance weight of hospitalization*

Using Eq.S12 and the result of section 1.4.4 such as Eq.S8, the small change in the expected number of hospitalizations after one generation is now written as

$$\frac{d\boldsymbol{h}\left( t+1 \right)}{d\boldsymbol{u}}=\frac{d\boldsymbol{Hx}\left( t+1 \right)}{d\boldsymbol{u}}=-\boldsymbol{H}\left( \boldsymbol{Q}_{S}\boldsymbol{N}^{-1}R\boldsymbol{w}_{1}\mathbf{v}_{1}^{\boldsymbol{T}}\boldsymbol{+}R\boldsymbol{w}_{1}\mathbf{v}_{1}^{\boldsymbol{T}}\boldsymbol{Q}_{T}\boldsymbol{N}^{-1} \right)\boldsymbol{x}(t).$$

Again, we define the projection matrix as $\boldsymbol{P}^{(H)}=\boldsymbol{H}\left( \boldsymbol{Q}_{S}\boldsymbol{N}^{-1}R\boldsymbol{w}_{1}\mathbf{v}_{1}^{\boldsymbol{T}}\boldsymbol{+}R\boldsymbol{w}_{1}\mathbf{v}_{1}^{\boldsymbol{T}}\boldsymbol{Q}_{T}\boldsymbol{N}^{-1} \right)$ and write ${\lambda_{1}}^{\left( H \right)}$ and $\boldsymbol{w}_{1}^{(H)}$ for the dominant eigenvalue and corresponding right eigen vector of $\boldsymbol{P}^{(H)}$. When the vaccination is targeted at the group *i*, the relative change in the dominant eigenvalue ${\lambda_{1}}^{(H)}$ is

$$\begin{aligned} {\lambda_{1}}^{\left( H \right)}w_{i1}^{(H)}=\left( \eta_{i}Rfg\left( q_{i}^{\left( S \right)}+q_{i}^{\left( T \right)} \right)\frac{c_{i}}{a_{i}}\frac{x_{i}\left( t \right)}{s_{i}\left( t \right)}\frac{x_{i}\left( t \right)}{n_{i}} \right)fx_{i}\left( t \right).\#\left( Eq.S13 \right) \end{aligned}$$

We define this expected impact of a single vaccination in group *i* on the dominant eigenvalue ${\lambda_{1}}^{(H)}$ as the importance weight of hospitalization:

$$\begin{aligned} y_{i}^{\left( H \right)}=\eta_{i}Rfg\left( q_{i}^{\left( S \right)}+q_{i}^{\left( T \right)} \right)\frac{c_{i}}{a_{i}}\frac{x_{i}\left( t \right)}{s_{i}\left( t \right)}\frac{x_{i}\left( t \right)}{n_{i}}.\#\left( Eq.S14 \right) \end{aligned}$$

We can interpret this quantity $y_{i}^{(H)}$ as the expected reduction in the number of new hospitalizations generated by a typical infected individual in group *i* after introducing a single unit of vaccine.

*1.6 Importance weights for other objectives*

We can replace the matrix $\boldsymbol{H}$ (i.e., a diagonal matrix with the elements of infection hospitalization rates per group *i*) with different rate matrices for other objectives. In this study, we also aimed to test an allocation strategy to minimize the number of deaths. Thus, we introduced a diagonal matrix $\boldsymbol{D}$ with group-specific infection mortality rate $\mu_{i}$ on the diagonal, and the importance weight of death $y_{i}^{(D)}$ can be derived in the same manner as the section 1.5;

$$\begin{aligned} y_{i}^{\left( D \right)}=\mu_{i}Rfg\left( q_{i}^{\left( S \right)}+q_{i}^{\left( T \right)} \right)\frac{c_{i}}{a_{i}}\frac{x_{i}\left( t \right)}{s_{i}\left( t \right)}\frac{x_{i}\left( t \right)}{n_{i}}.\#\left( Eq.S15 \right) \end{aligned}$$

We can interpret this quantity $y_{i}^{(D)}$ as the expected reduction in the number of new deaths generated by a typical infected individual in group *i* after introducing a single unit of vaccine.

*1.7 Changes in importance weights during the allocation of vaccines*

Since importance weights are dependent on the number of allocated vaccines, we need to update them at each allocation step. We denote the changes in importance weights in group *i* per single allocation as $\frac{dy_{i}^{(I)}}{du_{i}}$, $\frac{dy_{i}^{(H)}}{du_{i}}$, and $\frac{dy_{i}^{(D)}}{du_{i}}$ for each objective.

When vaccines are allocated, the eigenvector $\boldsymbol{w}_{1}$ is perturbed, and its small change $\frac{d\boldsymbol{w}_{1}}{d\boldsymbol{u}}$ can be approximated using power iteration with the new matrix $\boldsymbol{K+}\frac{d\boldsymbol{K}}{d\boldsymbol{U}}$ (see [7], p.331):

$$\boldsymbol{w}_{1}+\frac{d\boldsymbol{w}_{1}}{d\boldsymbol{u}}\sim\left( \boldsymbol{K+}\frac{d\boldsymbol{K}}{d\boldsymbol{U}} \right)\boldsymbol{w}_{1}\propto\boldsymbol{K}\boldsymbol{w}_{1}+\frac{d\boldsymbol{K}}{d\boldsymbol{U}}\boldsymbol{w}_{1}\propto R\boldsymbol{w}_{1}-\boldsymbol{Q}_{\boldsymbol{s}}\boldsymbol{N}^{-1}R\boldsymbol{w}_{1}$$

where the sign $\propto$ means “proportional to” and the sign $\sim$ means “approximately proportional to”. The same derivation has been introduced elsewhere (see section 3.6. of supporting info in [5]). If the allocation of vaccines is targeted at the group *i*, this results in the change in the *i* th element of the top right eigenvector, such as

$$\begin{aligned} w_{i1}+\frac{dw_{i1}}{du_{i}}\sim w_{i1}-\frac{q_{i}^{\left( S \right)}}{n_{i}}w_{i1}\#\left( Eq.S16 \right) \end{aligned}$$

And all other elements remain unchanged [5]. We use this equation to quantify the changes in importance weights after the allocation of a single unit of vaccines. By multiplying both sides of Eq.S16 by the factor $Rg\left( q_{i}^{\left( S \right)}+q_{i}^{\left( T \right)} \right)\frac{c_{i}}{a_{i}}\frac{x_{i}\left( t \right)}{s_{i}\left( t \right)}\frac{1}{n_{i}}$ and using $w_{i1}\approx fx_{i}\left( t \right)$, from Eq.S2 and Eq.S11, we obtain

$$y_{i}^{(I)}+\frac{dy_{i}^{(I)}}{du_{i}}\sim y_{i}^{(I)}-\frac{q_{i}^{\left( S \right)}}{n_{i}}y_{i}^{(I)}.$$

Subsequently, we can define the change in the importance weights of hospitalization and death. By multiplying both sides of Eq.S16 by factors $\eta_{i}Rg\left( q_{i}^{\left( S \right)}+q_{i}^{\left( T \right)} \right)\frac{c_{i}}{a_{i}}\frac{x_{i}\left( t \right)}{s_{i}\left( t \right)}\frac{1}{n_{i}}$ and $\mu_{i}Rg\left( q_{i}^{\left( S \right)}+q_{i}^{\left( T \right)} \right)\frac{c_{i}}{a_{i}}\frac{x_{i}\left( t \right)}{s_{i}\left( t \right)}\frac{1}{n_{i}}$ respectively, from Eq.S2 and Eq.S14-15, we obtain

$$y_{i}^{(H)}+\frac{dy_{i}^{(H)}}{du_{i}}\sim y_{i}^{(H)}-\frac{q_{i}^{\left( S \right)}}{n_{i}}y_{i}^{(H)}$$

and

$$y_{i}^{(D)}+\frac{dy_{i}^{(D)}}{du_{i}}\sim y_{i}^{(D)}-\frac{q_{i}^{\left( S \right)}}{n_{i}}y_{i}^{(D)}.$$

The perturbation due to vaccination in group *i* does not affect other groups under the condition that $\boldsymbol{K}$ is approximated.

**Reference**

1. Inaba H. Age-Structured Population Dynamics in Demography and Epidemiology. Springer; 2017.

2. Caswell H. Sensitivity Analysis: Matrix Methods in Demography and Ecology. Springer, Cham; 2019.

3. Magnus JR, Neudecker H. Matrix Differential Calculus with Applications in Statistics and Econometrics. Wiley; 1988.

4. Diekmann O, Heesterbeek JAP. Mathematical Epidemiology of Infectious Diseases: Model Building, Analysis and Interpretation. John Wiley & Sons; 2000.

5. Wallinga J, van Boven M, Lipsitch M. Optimizing infectious disease interventions during an emerging epidemic. Proc Natl Acad Sci U S A. 2010;107: 923–928.

6. Caswell H. Matrix population models. Sinauer Sunderland, MA, USA; 2000.

7. Golub GH, Van Loan CF. Matrix computations (3rd ed.). USA: Johns Hopkins University Press; 1996.
